# A multi-faceted pandemic: a review of the state of knowledge on the Zika virus

**DOI:** 10.1186/s40985-018-0087-6

**Published:** 2018-05-15

**Authors:** Anneliese Depoux, Aline Philibert, Serge Rabier, Henri-Jean Philippe, Arnaud Fontanet, Antoine Flahault

**Affiliations:** 1 Centre Virchow-Villermé, Université Sorbonne Paris Cité, Paris, France; 2 GRIPIC, EA 1498, Université Paris Sorbonne – CELSA, Paris, France; 3 Interdisciplinary Research Centre on Well-being, Health, Society and Environment (Cinbiose), Université du Québec à Montréal, Montreal, Québec Canada; 4 Faculté de Médecine, Université Paris Descartes, Paris, France; 5 Service de Chirurgie Générale, Plastique et Ambulatoire, AP-HP, HUPC, Hôpital Cochin, Paris, France; 6 Unité PACRI, Conservatoire National des Arts et Métiers, Paris, France; 7 Institut Pasteur, Emerging Diseases Epidemiology Unit, Paris, France; 8 Institute of Global Health, Faculty of Medicine, University of Geneva, Geneva, Switzerland

**Keywords:** ZIKV, Neurologic disorders, Vector control, Microcephaly, *Aedes aegypti*, MOOC, Health promotion, Health policies, Health management, WHO

## Abstract

While until recently the small and isolated Zika outbreaks in Eastern Asia and Pacific islands had been overlooked, the large-scale outbreak that started in Brazil in 2015 and the increase of microcephaly cases in the same place and time made media headlines. Considered as harmless until recently, Zika has given rise to an important global crisis that poses not only health challenges but also environmental, economical, social, and ethical challenges for states and people around the world. The main objective of this paper is to review the recent Zika outbreak by covering a broad range of disciplines and their interactions. This paper synthetises experts’ interviews and reactions conducted during a Massive Open Online Course (MOOC) entitled “In the footsteps of Zika…approaching the unknown.” It reviews knowledge and uncertainties around epidemiology, geographical dispersion of the virus and its vectors through globalization and climate change, and also its modes of transmission, diagnosis, symptoms, and treatment of the disease. The resulting societal and ethical issues in pregnancy and women of reproductive age were also addressed as well as the global outbreak alert and response network in international organizations and social media. This paper attempted to combine each piece of the jigsaw puzzle of the Zika phenomenon to complete the best realistic picture, while keeping in mind the balance between the interdisciplinary nature and international context of Zika and its unique characteristics.

## Background

Although the Zika virus (ZIKV) has been around for decades, it was seen as a rare and relatively benign infection until recently [1, 2]. It was not until 2007 after a Zika outbreak hit the Pacific islands in Yap (the Federated States of Micronesia) and French Polynesia a few years later (2013–2014) that the research community devoted more attention to the virus [1]. From the end of 2015 onwards, ZIKV received worldwide attention when it spread explosively in Brazil accompanied by a spike in microcephalic births. It turned into a public health emergency of international concern in early 2016 [3, 4].

Although transformation from a sleeping virus to a global crisis has become a familiar narrative with the reemergence of many “sleeping epidemics” such as the dengue fever in 2001, Chikunguna in 2004, or Ebola in 2014, the distinctive feature of the Zika outbreak lies in its astonishingly rapid geographical expansion and its unusual threat to the so far unaffected industrialized nations, through ever-accelerating urbanization and globalization. The worldwide concern generated by the increasingly globalized Zika phenomenon has revealed the various impacts of the outbreak on society. Indeed, the impacts that go beyond health are usually ignored, especially when they occur in remote (sub)tropical areas and/or low-income countries. An interdisciplinary approach provides a great opportunity to state widespread implications of the Zika virus through a number of different social impacts. Based on expert’s interviews conducted during a Massive Open Online Course (MOOC) entitled “In the footsteps of Zika… approaching the unknown,” the main objective of this paper is to review knowledge and uncertainties on the recent Zika phenomenon by covering a range of disciplines as follows. The paper will include epidemiology, historical and geographical dispersion of the virus and its vectors through globalization and climate change, its modes of transmission, and also diagnosis and treatment. The different types of preventive measures will also be covered. A specific section will be dedicated on neurological adverse effects such as the risk for birth defects (microcephaly) in pregnant women and peripheral nerve paralysis called Guillaume-Barré syndrome. The fact that Zika outbreak has reinforced social inequity and ethical issues through South America will be addressed. The resulting revolution in cultural, religious, and legal norms in the face of the expansion of Zika will also be discussed. Finally, the present work will state the recommendations and response measures of policy makers and public health organizations and social media.

## Methods

This paper reviews information gathered in a MOOC entitled “In the footsteps of Zika… approaching the unknown” (available on the platform coursera: www.coursera.org). This MOOC brought together 35 faculty members and international experts to foster the understanding of the stakes concerning Zika pandemics. Based on an interdisciplinary approach, the MOOC successively addressed a range of topics in eight modules. Each module was organized around a central topic to develop participants’ knowledge and skills. Modules were comprised of interviews with specialists, discussions and interactions, quizzes, recommended readings, online videos, and web links.

The present paper carefully extracts the content of interviews and combines information into eight sections presented as follows. The “Introduction to the Zika virus, its vectors, and its hosts” and “Following the tracks of Zika virus” sections introduce the origins of ZIKV, as well as its vectors and hosts. These sections also aim to address how climate change, including the El Niño/La Niña phenomenon, might influence the propagation of the virus in various parts of the globe. The “Prevention and control” section reviews the prevention and control measures deployed to contain the emerging pandemic. In addition to traditional vector control programs, such as the use of insecticides, new ecological techniques are also discussed. In the “Clinical manifestations of Zika virus disease” section, special attention is paid to the clinical manifestations of the Zika virus disease and especially to the adverse neurological effects of the virus, such as microcephaly in fetus and Guillain-Barré Syndrome among adults. This section also discusses the key uncertainties that remain with regard to such adverse effects. The “Laboratory diagnosis of Zika infection and treatment” section addresses the laboratory diagnosis of Zika infection and possible treatments. Aside from the risk of microcephaly, the “Inequality faced with Zika exposure and outcomes” section explores the inequalities existing between social classes during the recent Zika outbreak, including differences in access to health care, socio-economic support, and reproductive rights. Finally, the “Duties and tasks of the World Health Organization” and “The role of the media in the prevention of the disease” sections review amount and type of information that has been circulated, while assessing the effectiveness of health promotion messages communicated by health organizations with the assistance of social media.

### Introduction to the Zika virus, its vectors, and its hosts

ZIKV is an emerging arthropod borne virus (arbovirus) belonging to the genus *Flavivirus* and the family *Flaviviridae*. It is closely related to other flaviviruses such as dengue (DENV), yellow fever, and West Nile and Japanese encephalitis. First discovered in 1947 from a sentinel rhesus macaque in the Zika Forest of Uganda during yellow fever surveillance [1], the first human cases of ZIKV were detected a few years later in Nigeria (1953) [5].

Since then, sporadic cases and small clusters of patients were reported in tropical Africa and Southeast Asia before emerging in the Pacific Islands in the late 2000 [4, 6]. The first reported major outbreak of Zika fever infected 5000 out of 6700 inhabitants on the Western Pacific island of Yap, in Micronesia (Western Pacific) in 2007 [6], followed by a larger epidemic in French Polynesia (South Pacific) with 28,000 persons (11% of the population) who sought medical care in 2013 and 2014. Two thirds of the population of 270,000 were infected in about 6 months by the virus [7]. By March 2015, the first suspected cases were reported in Brazil, and ZIKV spread rapidly throughout the Americas [4]. Some phylogenetic studies indicate that the virus that spread in the Americas was significantly similar to African genotypes but was most closely related to the Asian strain that circulated in French Polynesia [8, 9]. It was hypothesized that the ZIKV introduction in French Polynesia from Asia spread throughout the South Pacific and entered Brazil in the late 2013 and/or in 2014, picked up at athletic events hosted in Pacific countries during the time when ZIKV was circulating [8, 9]. In early 2016, the microcephaly outbreak triggered an international public health emergency following a 20-fold increase in human birth defects (WHO, Feb 1, 2016) [10]. In late September 2017, the Pan-American Health Organization (PAHO) estimates that around 138,000 confirmed infections (non-confirmed cases 232,000) have occurred in Brazil since the beginning of the outbreak [11]. Due to the spread of ZIKV in many other countries in South and Central America and the Caribbean, more than 750,000 suspected and confirmed cases were reported in this region by late September 2017 (around 580,000 suspected versus 221,000 confirmed cases) [11]. Today, ZIKV continues to spread geographically through the Americas, to areas where vector competence of vector mosquito species (ability for infection, dissemination, and transmission of virus) is high [12, 13]. From the 70 countries or territories worldwide that have found evidence of mosquito-borne ZIKV transmission since 2015, a total of 55 have reported ZIKV outbreaks, of which 48 are located in the Americas and Carribean regions [11]. By March 2017, a total of 13 countries had reported evidence of person-to-person transmission of Zika virus (7 in Europe, 5 in the Americas, and 1 in the Pacific) [11].

Although travelers returning from areas with endemic/epidemic Zika fever may initiate local ZIKV transmission and potentially sporadic cases in non-endemic areas, the primary method of transmission remains the bite of an infected female mosquito, especially of the genus *Aedes* [14]. Because mosquitoes are hematophagous arthropods, they acquire the virus via a blood meal and host it throughout their life-span without being affected [15]. Some *Aedes* species have a limited distribution (e.g., *Aedes luteocephalus* in Africa and *Aedes hensilli* in the Pacific Islands), while others have a broad geographic range such as *Aedes albopictus*, which can exist in more temperate areas [15, 16]. Although routes of human transmission other than the mosquito exist, such as sexual intercourse (horizontal transmission) [17, 18], perinatal transmission from mothers to fetus (vertical transmission) [19] and post-transfusion transmission [20], they have not shown the potential for maintaining ZIKV transmission. It was clearly shown that frequency and duration of detectable ZIKV RNA varied between human body fluids [21]. The median times until the loss of ZIKV RNA detection were 14 days in serum, 8 days in urine, and 34 days in semen, with maximum time up to 6 months for the latter [17, 18, 20].

### Following the tracks of Zika virus

The Zika virus left the remote African forests for new international destinations primarily because of globalization and climate change. Zika was an example of an animal pathogen making the leap from monkey to human host in Uganda, where humans were forced into close proximity with animals. The virus spread mostly through movements of infected humans, and air travel has dramatically sped up the process in the last decades. This in turn presents pathogens with a novel pathway of rapid spread as infected individuals may be unaware of an illness before arriving in their new locale. This has almost certainly facilitated the geographic range expansion of numerous human pathogens including Zika virus. These translocated diseases find another dispersal aid in the shape of our rapidly exploding population. Overcrowding, strained public services and sanitation challenges all facilitate the increased speed of disease transfer through host populations. Given that the Zika virus is mainly transmitted by *Aedes* mosquitoes, its outbreak potential is heavily influenced by climate and environmental conditions [22]. Flood events often precede a malarial outbreak and are a major predictor of malaria. However, the opposite is true for a number of other mosquito-borne diseases: Zika, dengue, yellow fever, and chikungunya are often triggered by drought and heat waves. In low income areas where homes are not equipped with tap water, people need to store water in close proximity to their houses during drought conditions, creating shelters for mosquitoes’ eggs. The link between climate, animal health, and human health is thus clear: using data on warming events, animal migration events, invasive species pathways, and droughts/floods, we can be better prepared to deal with impending disease outbreaks. The El Niño phenomenon, which is a South Pacific climate oscillation, may result in a decrease in rainfall associated with heat waves in some parts of the world, such as Northeastern Brazil. This region is amongst the poorest of the country with limited access to urban infrastructure and tap water [23] despite recent improvements. In addition, although they are still controversial, there are many arguments in favor of an increased risk of a more frequent and more intense El Niño/La Niña phenomenon due to climate change [24]. This may play a leading role in the emergence of outbreaks of arboviruses in those parts of the world which are most affected by its influences on precipitation and temperature. Satellite data and predictive models suggested high potential for an outbreak of dengue fever in 2015 in Brazil but the relevant authorities failed to respond adequately to the threat, and a Zika outbreak occurred because the same mosquitoes that carry dengue also carry Zika. This link however was not made at the time and the risk not fully understood.

### Prevention and control

Although numerous strategies for the prevention of vector exposure already exist (repellents, window screens, mosquito nets, long clothing), they have limited efficacy against *Aedes* mosquitoes, which bite during the day. Additionally, mosquito-control strategies have proved inadequate in containing viruses through the removal of breeding sites or by mosquito suppression. While the use of chemical insecticides has been the largest component over the decades to control *Aedes* mosquito populations (*Aedes aegypti*), excessive use has led to widespread resistance in mosquitoes [25] and may have environmental consequences. Recently, new interest has been devoted to the potentially more eco-friendly control of mosquito vectors called *Wolbachia*. *Wolbachia pipientis* is an intracellular bacterium or symbiont found in a wide range of arthropod species [26, 27]. It protects its hosts (mosquitoes) against pathogens (including arboviruses, bacteria, filarial nematodes and the malaria parasite, *Plasmodium gallinaceum*) by limiting their growth and eventually their transmission to humans [26, 27]. Although the protection process is not fully understood yet, previous work suggests that *Wolbachia* activates the immune response of its host, competes for host resources critical for viruses, and manipulates the host viral defense through the microRNA pathway. Laboratory results showed that *Wolbachia* infection reduces viral replication of several viruses such as dengue, chikungunya, and ZIKV in *Aedes* mosquitoes and eliminates or delays the appearance of the virus in mosquito saliva, reducing its efficacy in transmitting viruses [27, 28]. This antiviral activity is considered to be stable and remains strong 1 year after field deployment. Because *Wolbachia* is known for its ability to spread rapidly through mosquito populations by manipulating the host’s reproduction to its advantage, the rationale behind this novel approach is that after the scale-up of deployment of *Wolbachia* in the field, it will pass into wild *Aedes aegypti* populations and eventually infect most of them. The *Wolbachia*-based strategy, as a multivalent strategy against *Ae. aegypti*-transmitted viruses, seems promising [28, 29].

### Clinical manifestations of Zika virus disease

The first known epidemic of Zika virus on Yap island, Micronesia, in 2007, provided an initial description of the clinical manifestations of the disease. Patients suffered from a mild disease, lasting a few days, consisting of a rash (90%), fever (65%), arthralgia (65%), conjunctivitis (55%), and other minor symptoms [7]. Moreover, following a post-epidemic serological survey, it was estimated that 80% of the infected individuals were asymptomatic. ZIKV infection was therefore perceived as an exotic curiosity, with very mild clinical manifestations when symptomatic. Six years later, when the ZIKV epidemic struck French Polynesia, a different clinical profile emerged. While most ZIKV cases were benign, physicians working at the Centre Hospitalier de Polynésie française in Papeete, Tahiti, noticed a steep rise in the number of patients referred for severe neurological conditions including Guillain-Barré syndrome (GBS), encephalitis, meningoencephalitis, and myelitis [30]. GBS, an autoimmune disease causing acute flaccid paralysis, was the most common and dramatic manifestation. Over the 6-month period of the epidemic, 42 patients were hospitalized with this condition, representing a 17-fold increase in incidence compared to previous years [7]. Disease evolution was rapid, with a median of 6 days from onset of symptoms to the plateau phase and then 4 days until the beginning of recovery. Still, 16 (38%) patients were transferred to the intensive care unit, mostly for respiratory assistance. All patients received intravenous immunoglobulin, and none of the patients died. Three months after hospital discharge, 24 (57%) patients were able to walk without assistance. Using a case-control design and serological testing, it was possible to show evidence of a link between ZIKV and GBS, although no consistent pattern of antiganglioside antibodies could be demonstrated. Following the worldwide expansion of ZIKV, 11 countries have reported an increase in GBS during outbreaks of ZIKV infection, and a review panel concluded that the most likely explanation for this trend is that ZIKV infection is a trigger of GBS [31].

The worst is still to come. On November 11, 2015, 6 months after the first reports of autochthonous transmission of ZIKV in the states of Bahia and Rio Grande do Norte, the Brazilian Ministry of Health declared a public health emergency following a substantial increase in the number of reported cases of congenital microcephaly compared to previous years [32]. Congenital microcephaly is a neurological abnormality that is present at birth and is usually defined as head circumference at least 2 SD smaller than the mean for sex and gestational age. The main causes of microcephaly are genetic aberrations or infections during pregnancy with cytomegalovirus, rubella, and toxoplasmosis, which lead to underdevelopment of the brain. The first solid epidemiological evidence of a link between ZIKV and microcephaly came from the follow-up of women in Rio de Janeiro with documented ZIKV infection during pregnancy [33]. This study and subsequent ones revealed that neurological abnormalities in fetuses/babies included not only microcephaly but also other manifestations such as seizures, irritability, arthrogryposis, brainstem dysfunction, ocular complications, and abnormal neuroimaging findings such as calcifications, hypogyration, and ventriculomegaly, defining what is now known as ZIKV congenital syndrome [34]. While there is no longer any doubt about the responsibility of ZIKV in these complications, a debate still exists surrounding their frequency; in Brazil, 46% of 125 fetuses/babies born to infected women had neurological abnormalities [33], as opposed to only 6% of 403 in the USA [35]. Likewise, in French Polynesia, retrospective analyses of medical abortions and births around the ZIKV epidemic period suggest that only 1% of women infected during the first trimester of pregnancy would have a child with microcephaly [36]. Current studies aim to identify local/environmental factors explaining the higher rate of complications in Brazil. In parallel to these epidemiological investigations, several in vitro and animal studies have documented the infection by ZIKV of neural progenitor cells and its impact on the brain development [37–39], confirming the earlier observation by G.W. Dick, who discovered a specific ZIKV tropism in mice brains [2].

In summary, ZIKV, with its potential for worldwide circulation in *Aedes aegypti* (and potentially *Ae. albopictus*) infested areas and further spread through sexual transmission, poses a new public health challenge, largely due to the congenital neurological complications that occur when women are infected during pregnancy.

### Laboratory diagnosis of Zika infection and treatment

Laboratory tests for Zika virus diagnosis are based on molecular and/or serological testing after clinical suspicion. ZIKV has a single-stranded RNA genome. Although viremia detection time will soon improve with novel technological advancements, ZIKV is actually captured in the serum during the acute phase of the illness, a few days before and after the onset for symptoms (the reported time limit varying between 7 and 28 days), while urine and saliva ZIKV detection reaches higher titer and lasts longer [21]. ZIKV has also been detected in many other body fluids such as semen, nasopharyngeal swabs, amniotic fluid, and breast milk [21].

Detection of ZIKV in body fluid is accomplished through molecular identification of viral RNA by reverse transcription-polymerase chain reaction (RT-PCR) or by other nucleic amplification tests (NAATs) [40]. If the acute phase, during which RNA detection is possible, is missed, diagnosis of ZIKV infection relies on antibody-based serological tests [41]. Such tests are less costly than RNA detection and do not require specialized reference laboratories. Immunoglobin (Ig) M-based serological tests may be positive from 7 days after disease onset up to several months, while IgG tests become positive a few days later and last for longer periods [40–42]. Confirmation can be performed by highly specific plaque reduction neutralization tests (PRNTs) [40]. However, serological tests present some limitations owing to their cross-reactivity with other flaviviruses such as dengue, and possibly Japanese encephalitis, Powassan, St. Louis encephalitis, West Nile virus, and yellow fever [42]. This is of concern for ZIKV diagnosis because dengue and yellow fever are likely to co-circulate in many areas, leading to uncertainties and/or difficulties to differentiate the ZIKV antibody responses from others [42]. In case of the congenital infection of the fetus, ZIKV screening may be performed in the amniotic fluid with RT-PCR assays. When an infant is born, IgM detection in cord blood or CSF may be the preferred method of detection. For pregnant women, RT-PCR in blood/urine performs well for diagnosing symptomatic infections [42], whereas seroconversion during pregnancy may detect asymptomatic infections, thus underlining the need for sensitive and specific immunoglobin (Ig)-based serological tests [41]. There has not yet been a specific medical treatment or a vaccine licensed for Zika virus (Centre for Disease Control and Prevention (CDC website) [43].

Efforts to develop a vaccine are moving fast [44]; however, it is not clear that the current epidemic will be sustained long enough for a phase III vaccine efficacy trial. In the absence of effective antiviral therapies or implemented vaccines against ZIKV, and considering the invasive behavior and ecological plasticity of Aedes mosquitoes, novel vector control approaches are receiving considerable attention in preventing and/or reducing ZIKV transmission [45].

### Inequality faced with Zika exposure and outcomes

Wealthier communities appear better prepared to face ZIKV than poorer ones in Brazil and South America. Aside from the increased risk of having a child with microcephaly, inequalities remain when accessing health care, socio-economical support, and/or reproductive rights [46]. Dramatic increases in Zika infections have occurred in more than 22 countries, in particular in Latin America, a continent where the influence of religious values both from the Catholic church (40% of the Catholics in the world live in Latin America according to the Agence Fides [47]) and from various expanding Evangelical Churches, sometimes even more radical in their teachings, strongly determine collective behaviors and individual gender expectations. The official doctrines of these institutions/organizations widely and explicitly prohibit or limit the rights to contraception and abortion and are entrenched in repressive domestic legal systems. Even when family planning services are a recognized constitutional right, as in Brazil [48], they remain largely inaccessible because of poverty and lack of education/information. Abortion is extremely limited: for example, in Brazil, abortion is possible only in cases of complications that endanger the mother’s life, rape, and fetal anencephaly. In practice, a recent study has shown that one in five women in Brazil, under the age of 40, had recourse to illegal abortions at least once in their life. Such abortions, which are sometimes carried out in non-sanitary, and therefore, dangerous conditions have led to increasing maternal mortality and morbidity rates. Only women in higher social classes can afford the cost (both psychologically and financially) of safe abortions. An overwhelming majority of women and adolescent girls, mostly people of color and poor, living in less developed areas, and subject to recurrent gender-based violence, had access only to unsafe abortions that were also deemed illegal. In such conditions of poverty, chronic sexual violence, denial of access to healthcare and facilities in reproductive matters, and their overall criminalization, the institutional and official recommendation given to women “to avoid or delay pregnancy” [49] appears to ignore the reality that (poor and uneducated) women and girls do not have the capacity and legitimacy to decide if, when and under what circumstances, they do not wish to become pregnant.

### Duties and tasks of the World Health Organization

Soon after the World Health Organization declared the ZIKV-related outbreak of microcephalies in Brazil a public health emergency of international concern in February 2016, it quickly launched a health promotion campaign using a wide range of communication tools. After the WHO was heavily criticized for its slow response react to the Ebola outbreak and its lack of understanding of the wider socio-economic factors that contributed to the epidemic spread, it decided to promptly respond and demonstrate leadership. This was especially important given that Brazil is a member of the G20 and was the host of the 2016 Summer Olympic Games in Rio de Janeiro. The WHO’s response to ZIKV is not just an emergency response, but rather a long-standing commitment. Indeed, although ZIKV and related neurological complications no longer represent a global health emergency, the WHO and its partners are committed long-term to updating the public with current and accurate information. The WHO has published situation reports for the 84 countries where the Zika virus was present and has coordinated the response of more than 60 local and global health partners [50].

### The role of the media in the prevention of the disease

The media plays a key role in social mobilization and in raising public awareness during health crises [51]. Network structures are crucial to synchronizing information on epidemics in the context of social networks. Digital media made an efficient effort to spread health promotion tools (including phone applications) and to gather useful data on the outbreak in order to measure the extent of the virus outbreak so as to enhance the response capacity of crisis managers [52].

At the beginning of 2016, when the Zika outbreak was raging in Brazil, the analysis of social network interactions and the quantitative evaluation of the questions initiated by web surfers were strong indicators to confirm two points: the extent to which media can mobilize people and the importance of announcements from public health authorities in order to generate the media coverage essential for awareness. Public health authorities and journalists are both a very important part of risks communication at a massive scale. Authorities give the alert, media spreads the message, and the population passes on the message through social networks. A study released in July 2016 [53], based on data produced in January and February 2016 (tweets and searches on Google in the USA, Guatemala, and Brazil), highlighted this point in the case of Zika. After the declaration of a public health emergency of international concern for ZIKV in Brazil and travel alerts from the CDC, especially advising pregnant women and their partners not to travel to outbreak areas, activity on social networks amplified significantly. This was also the case on February 3, 2016, when authorities announced the first autochthonous case of infection in the USA.

The use of social networks to reach a broader population is one of the approaches identified by public health authorities to address health crises identified by the WHO, the US CDC, and Ministries of health, among others. In its action plan against Zika, the US CDC recommends a proactive use of all communication channels, and particularly social networks, to deliver key messages in terms of risks, prevention, diagnosis, and care [54]. The WHO has developed an application for mobile phones, which gives access to a variety of resources and sent messages of prevention and early warning information [55].

Online media like Facebook or Twitter provide a very large diffusion of information through different communities, which we also call virality. On February 2, 2016, in the first weeks of the outbreak, Mark Zuckerberg, the creator of Facebook, the most globally used social network, committed to fighting Zika through media virality. Facebook, in partnership with the non-profit Brazilian organization, Abrasco Divulga, produced a short video to alert pregnant women about risks linked to the Zika virus [56]. This video, which was watched more than a million times, shows a new kind of commitment in which the media appears as an actor of prevention by using its own tools as a broadcast channel. It is part of a wider logic, the renewal of philanthropy: charity takes a new path. Google has also taken part in this new philanthropic path, if it can be described that way. In addition to a financial donation to UNICEF, it provided tools enabling the visualization of the spread of the outbreak in real time. Creating an IT platform, an open opportunity to analyze various data, Google wanted to help by giving states and NGOs useful information to introduce very specific prevention actions, which are, therefore, more efficient.

## Conclusion

By adopting an interdisciplinary approach that incorporated different disciplines and experts, this paper attempted to combine each small piece of the puzzle to complete a realistic picture of the Zika phenomenon. Our main challenge with such an integrative approach was not only to reach a balance between the complex and multidimensional nature of Zika and its unique qualities but also to accept the iterative character of the learning process, which evolved with both increasing knowledge and persistent uncertainties. It is therefore no coincidence that long-term interdisciplinary collaborations all around the world have emerged to gather integrative knowledge in order to build a global approach to prevention and preparedness to the occurrence of viral outbreaks. It is time to connect the dots between a range of topics such as entomology, environment, climate change, and health and socioeconomical aspects, to name just a few.
